# Enhancing the Trajectory Generation of a Stair-Climbing Mobility System

**DOI:** 10.3390/s17112608

**Published:** 2017-11-13

**Authors:** Jose Abel Chocoteco, Rafael Morales, Vicente Feliu-Batlle

**Affiliations:** 1 Instituto Tecnológico de Ciudad Guzmán (ITCG), Tecnológico Nacional de México (TecNM), Ciudad Guzmán 49100, Mexico; jachocoteco@itcg.edu.mx; 2 School of Industrial Engineering, University of Castilla-La Mancha (UCLM), 02071 Albacete, Spain; 3 School of Industrial Engineering, University of Castilla-La Mancha (UCLM), 13071 Ciudad Real, Spain; Vicente.Feliu@uclm.es

**Keywords:** architectural barriers, stair-climbing mobility system, trajectory generation, assistive technology

## Abstract

Recent advances in mobile robotic technologies have enabled significant progress to be made in the development of Stair-Climbing Mobility Systems (SCMSs) for people with mobility impairments and limitations. These devices are mainly characterized by their ability to negotiate those architectural barriers associated with climbing stairs (curbs, ramps, etc.). The development of advanced trajectory generators with which to surpass such architectural barriers is one of the most important aspects of SCMSs that has not yet been appropriately exploited. These advanced trajectory generators have a considerable influence on the time invested in the stair climbing process and on passenger comfort and, consequently, provide people with physical disabilities with greater independence and a higher quality of life. In this paper, we propose a new nonlinear trajectory generator for an SCMS. This generator balances the stair-climbing time and the user’s comfort and includes the most important constraints inherent to the system behavior: the geometry of the architectural barrier, the reconfigurable nature of the SCMS (discontinuous states), SCMS state-transition diagrams, comfort restrictions and physical limitations as regards the actuators, speed and acceleration. The SCMS was tested on a real two-step staircase using different time-comfort combinations and different climbing strategies to verify the effectiveness and the robustness of the proposed approach.

## 1. Introduction

Stair-Climbing Mobility Systems (SCMSs) are assistive devices for people whose mobility is impaired and limited. These devices are mainly characterized by their ability to negotiate those architectural barriers associated with climbing stairs. In this respect, SCMSs whose locomotion systems combine more than one mechanism of a different nature have a greater ability to surpass stairs than other models. These SCMSs, which are known as hybrid SCMSs, generally combine wheeled and legged systems (see, for example, [1,2,3,4,5]), wheel clusters attached to powered linkages (see, for example, [6,7,8]) and wheeled and tracked systems (see, for example, [9,10,11,12]). However, despite the fact that hybrid SCMSs are the most suitable systems with which to surpass stairs, they still have problems as regards guaranteeing the user a high factor of safety and comfort, signifying that most are not completely autonomous and external assistance is therefore required (see recent reviews by [13,14]).

Hybrid SCMSs can be divided into two major categories: semi-autonomous and fully-autonomous. Semi-autonomous SCMSs require a great deal of user interaction to surpass stairs (in some cases, external assistance, such as another person or a fixed structure, is required to surpass the obstacle). Semi-autonomous SCMSs are characterized by the fact that their stair-climbing modes are not based on trajectory planning. Abrupt movements or collisions usually occur because the users do not control the SCMSs correctly. In this case, the climbing process may be uncomfortable for the passenger, and security cannot be guaranteed without appropriate assistance. Two examples of this type of SCMS are the iBOT mobility system [8] and the TopChair [9]. Unlike semi-autonomous SCMSs, fully-autonomous SCMSs require minimal user interaction and do not require external assistance when negotiating stairs. With fully-autonomous SCMSs, a trajectory planning is considered for the stair-climbing operation. These models generally utilize algorithms to detect objects and avoid collisions and, in some cases, algorithms with which to estimate the dimensions of the stairs in order to derive a path using trajectory planning and trajectory optimization strategies. Little research in the literature deals with trajectory planning and trajectory optimization for fully-autonomous SCMSs. Three examples of this type of SCMS are presented in [4,15,16]. These fully-autonomous SCMSs employ strategies that allow them to know the environment (they estimate the size of the stairs using laser sensors).

Electric Powered Wheelchair (EPW) comfort has been identified as a priority in many studies, some major examples of which include [17,18,19,20,21,22,23,24,25,26]. Yet, there seems to be little agreement among ergonomists or disability researchers as how best to quantify discomfort or produce mechanisms that can reliably link feelings of discomfort with qualitative indicators. Some indicators and criteria regarding the assessment of human comfort in electric powered wheelchairs are proposed in [27,28,29,30,31,32]). Something similar occurs with current SCMSs, which do not adequately meet the need for comfort, mainly during the stair-climbing task. Relatively few research efforts have, therefore, been focused on the discomfort associated with SCMSs, although three recent examples of this include [5,33,34].

**Figure 1:**
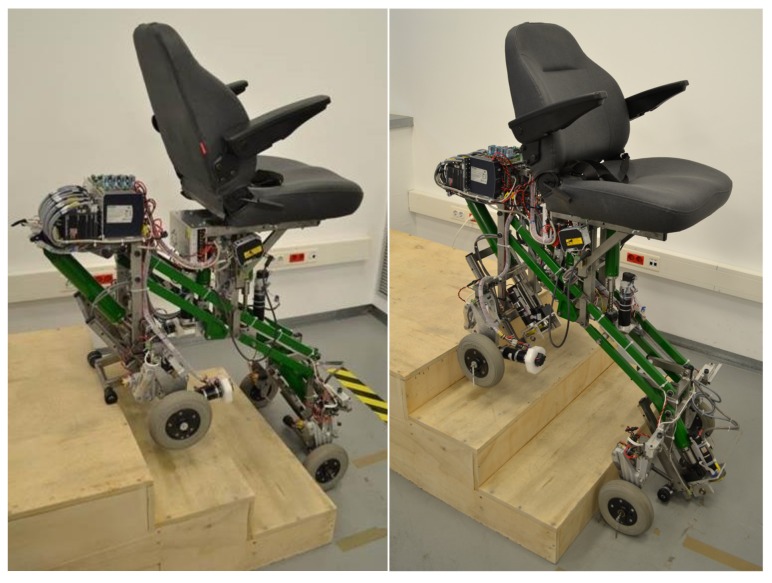
The proposed Stair-Climbing Mobility System (SCMS) is characterized by the fact that it can climb up and down stairs of different sizes and maintain a stable equilibrium on the stair at all times without assistance. It is also characterized by the fact that it can estimate the size of the stair using laser distance sensors and generate safe and comfortable trajectories for the central platform when carrying a user.

In this paper, we propose a trajectory planning approach based on the optimization of both stair-climbing execution time and user comfort for our fully-autonomous SCMS, which is shown in Figure 1. We propose to deal with the problem of generating optimal and smooth trajectories under various kinematic and dynamic constraints by using a simple numerical method. The trajectory generation problem is formulated as a nonlinear optimization problem by parameterizing both the SCMS path and the associated motion profile using a set of control points which are fitted by means of B-spline functions [35,36]. These control points are directly extracted from a reference trajectory initially based on the current knowledge of the size of the stair. Trajectory planning with which to control the position of the center of mass of the SCMS, denoted as $P_{g}$, can then be performed using smooth trajectories, which have to be continuous and at least twice differentiable. The objective of the proposed method is to minimize a cost function, which is a weighted balance of stair-climbing time and the user’s comfort. This optimization problem is solved by using a Sequential Quadratic Programming (SQP) technique. Moreover, the behavior of the SCMS and the maintenance of the vertical position of its chassis are notably improved when using the proposed trajectory planning. Experiments have been carried out in order to evaluate the efficiency of the proposed approach.

The remainder of the paper is organized as follows: Section 2 presents a brief description of the mechanical design (mechanism and operating modes) and the environment recognition of the reconfigurable SCMS. Section 3 is devoted to the description of the kinematics and dynamics of the SCMS. The statement of the trajectory generator is depicted in Section 4. Section 5 briefly describes the experimental platform and the experiments performed to demonstrate the robustness of the proposed approach by carrying out several trials with different time-comfort combinations and different climbing strategies. Finally, Section 6 is devoted to the conclusions of the work.

## 2. Experimental System

The SCMS presented in this paper was built by a team of researchers at the School of Industrial Engineering at the UCLMuniversity. The SCMS developed is considered to be a hybrid SCMS since it combines two different types of locomotion to solve the stair-climbing problem. The SCMS uses wheeled and sliding support systems.

### 2.1. Mechanical Description

**Figure 2:**
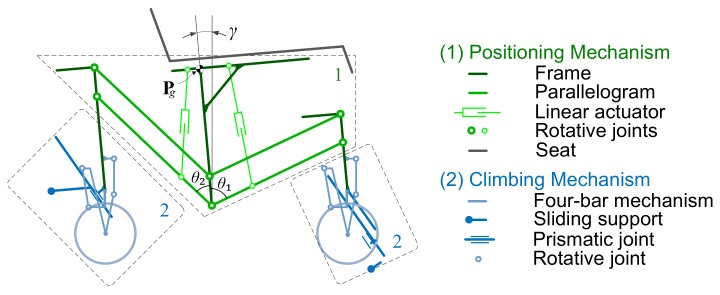
Kinematic scheme of the SCMS: (1) positioning mechanism, which is responsible for ensuring the posture of the entire vehicle; (2) climbing mechanism, which is responsible for surpassing the obstacle.

The general design of the SCMS is based on splitting the obstacle-climbing problem into two different problems of a lower order of difficulty. These problems are: (1) posture control of the vehicle and accommodating the wheels to the staircase; and (2) traction control of the wheels and surpassing the step. Each problem is solved by using two independent mechanisms; the first problem is solved by using a positioning mechanism while the second is solved by using two similar climbing mechanisms (see Figure 2). These latter mechanisms are those which combine the aforementioned wheeled and sliding support systems. A key aspect of the mechanical design is that both mechanisms can act independently of each other during the locomotion, which makes it possible to use different stair-climbing strategies.

The design of the positioning mechanism is based on a closed-loop mechanism. This mechanism is composed of three frames: a central one on which the seat where the user sits is placed and a rear and a front one to hold the proposed climbing mechanisms. The frames are connected to each other by two parallelograms that prevent relative rotation occurring between them. The central frame is also called the SCMS frame. Its inclination with regard to the direction of gravity is the controlled variable and is represented using γ. The positioning mechanism needs four stable support points at each instant to maintain the SCMS posture and to position the vehicle wheels on the staircase. These points are supplied by the two climbing mechanisms by means of their four wheels and two sliding supports, two wheels and one support for each mechanism. The positioning mechanism has two Degrees of Freedom (DOFs), each of which is driven by a linear actuator. The positioning mechanism can adapt to different stair geometries, which includes positioning both axles in order to maintain the stability and accommodating the front wheels on the staircase.

The climbing mechanisms are designed to surpass curbs or stairs. Our hybrid SCMS has two similar and independent climbing mechanisms; one front and the other rear. Each mechanism combines two wheels, one on each side, and one sliding support. The driving wheels are coupled to the rear climbing mechanism and they are controlled by direct current motors. The sliding supports are the supports in charge of surpassing the obstacles and they are controlled by linear actuators, similar to those used in the positioning mechanism. These mechanisms make it possible for the SCMS to surpass different sized stairs. The climbing mechanism combines four-bar mechanisms to control the wheels when the SCMS confronts the steps. These mechanisms are designed to be used with either the front or the rear wheels. This design also enables the same control technique to be used for all the wheels.

**Figure 3:**
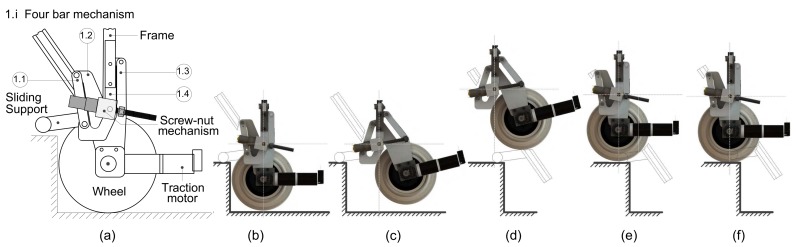
Actuating sequence of the rear climbing mechanism. . In (**a**) the wheel makes contact with the ground. In (**b**) the sliding support slides and makes contact with the step tread and the wheel is off the ground. In (**c**) the screw-nut mechanism is again actuated and the sliding support continues to slide. In (**d**) the sliding support is completely deployed. In (**e**) the screw-nut mechanism is actuated and the wheel moves backwards to its initial point. In (**f**) the wheel again makes contact with the ground and the sliding support is retracted.

The operation of the proposed climbing mechanism when climbing a step is outlined in the sequence of pictures in Figure 3. The weight is initially supported by the wheel, which rolls up closer to the step. It stops when it is close to the stair riser (Figure 3a). The sliding support is deployed until the instant at which the wheels are off the ground, as shown in Figure 3b. sliding support makes contact with the step tread. The wheel then moves upwards to climb the step in order to avoid interference with the corner of the step (Figure 3c). When the step has been surpassed (Figure 3d) the wheel moves backwards to its initial point, which is referred to as the frame (Figure 3e). The sliding support continues to maintain contact with the step tread while the wheel climbs. Finally, the support is retracted (Figure 3f). This last operation is performed in order to once again transfer the weight from the sliding support to the wheel. The descending operation runs similarly and conversely to the ascending operation. During the climbing/descent process, the wheels do not need traction since the movement and stability are guaranteed by the sliding support, which assumes the greatest responsibility for the obstacle being surpassed. The positioning mechanism operation is independent of the wheels while the climbing mechanism climbs/descends an obstacle. This latter mechanism can negotiate stairs of different sizes, even with geometric disturbances, and maintain a stable equilibrium on the staircase at all times.

### 2.2. Operating Modes

**Figure 4:**
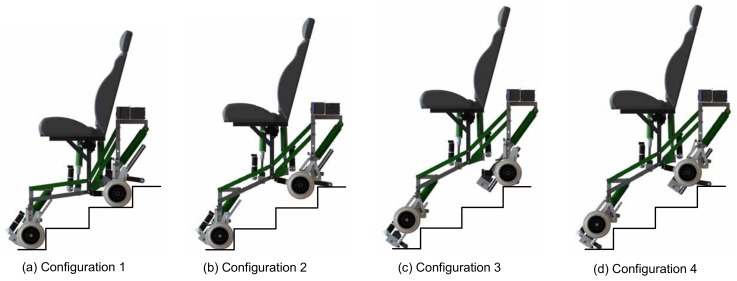
Possible configurations of the SCMS.

The SCMS has four possible operating modes, which we call configurations (see Figure 4). Configuration 1 (the most frequently used) is performed when the device is supported on four wheels. This configuration can be used for several purposes: (1) moving not only on level terrain, but also on tilted terrains, such as operating an usual EPW; (2) adjustment of the seat height, thus allowing the user to move around at eye level or/and reach items on high shelves and; (3) surpassing typical architectural barriers such as curbs, ramps or staircases. Configurations 2 and 3 are performed when the device is supported on the front wheels and the rear sliding support and the rear wheels and the front sliding support, respectively. These configurations are used to surpass curbs or staircases. Finally, Configuration 4, which is performed when the device is supported on the two sliding supports, is used only to surpass stairs with more than two steps.

### 2.3. Laser Distance Sensors for Environment Recognition

Two laser distance sensors are used to perform the strategy employed to estimate the size of the stair. These sensors (BOD 63MLA04-S115) are manufactured by Balluff. They have a working range of between 200 mm and 6000 mm; a nominal response time of less than 2 ms; and a nominal resolution of less than 1 mm. This type of sensors works on the principle of Time-Of-Flight (TOF) measurement, which is an excellent cost-effective tool for many modern mapping problems. All the technical specifications and characteristics of these sensors are described in [37].

**Figure 5:**
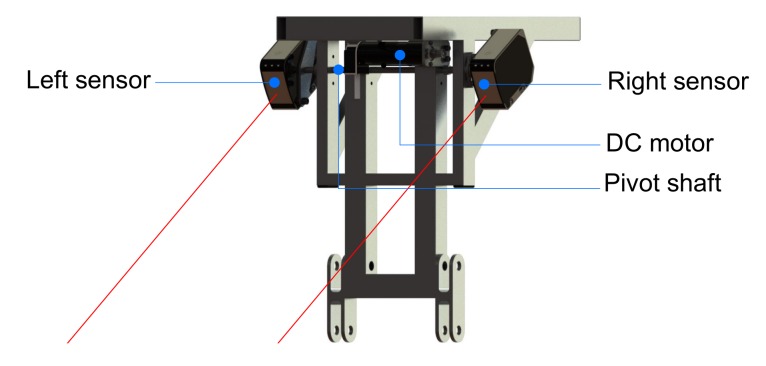
Scanning system positioned on the SCMS frame.

The sensors are strategically positioned on the lateral parts of the central frame of the SCMS (see Figure 5). They are mechanically coupled to a pivot shaft, which is powered by a DC motor with a belt-pulley transmission. The motor (Maxon Motor 226774, maxon motor ag, Sachseln, Switzerland) combines a gearbox (Maxon Motor 166174, reduction 246:1, maxon motor ag, Sachseln, Switzerland) and an optical incremental encoder with which to sense position. The motor is controlled by means of an EPOS2 positioning controller (maxon motor ag, Sachseln, Switzerland).

**Figure 6:**
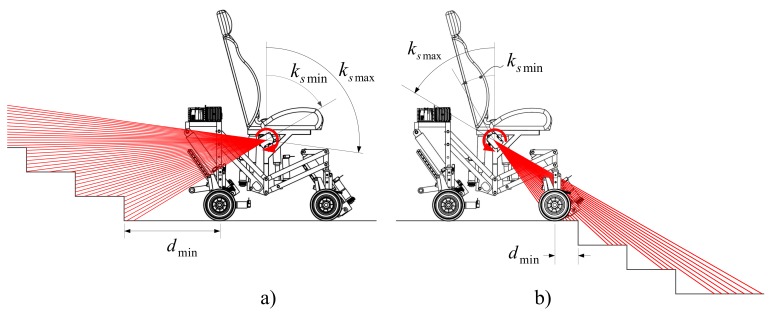
Side view of scanning on the staircase surface; (**a**) before climbing up and (**b**) before climbing down.

From the sensors’ strategic position, the architectural barrier can be scanned in both backward and forward directions without the chassis interfering with the laser pointers. They point backward when climbing up and forward when climbing down (see Figure 6). In addition, thanks to this strategic position, the system does not need any additional sensors to detect obstacles or to avoid obstruction during either the climbing or the descent processes. The estimation strategy greatly simplifies the control and sensory systems.

The estimation is obtained using a geometric analysis based on the scanning results and the odometry data. Figure 6a shows the scanning of the staircase surface that takes place before climbing up. The scanning is performed by rotating the two laser distance sensors in a clockwise direction. The angular position of the sensors is defined by the angle $k_{s}$. The field of view of the sensors allows the laser beams to project on the steps’ surfaces without interference from the SCMS chassis. The scanning is executed only once, and this is sufficient to perform the geometric analysis and obtain the stairs’ dimensions. In practice, the SCMS must carry out a new scan every four steps.

**Figure 7:**
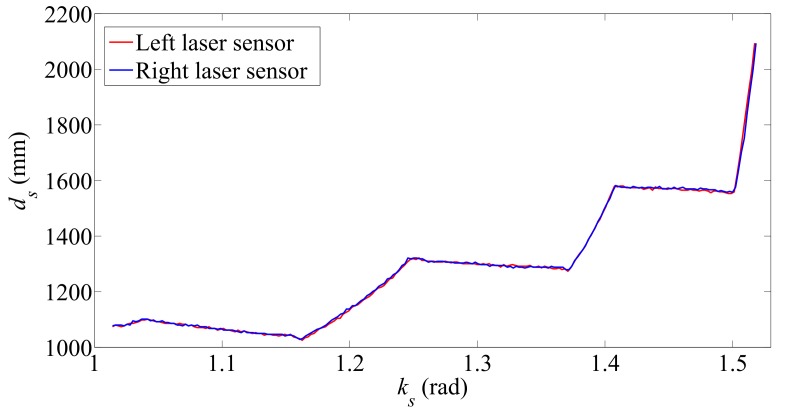
Distance measured during the scanning on the staircase surface before climbing up.

Figure 7 shows the results obtained from the scanning of the staircase surface before climbing up. The overlapping of the resulting trajectories demonstrates that the SCMS is aligned with the staircase.

**Figure 8:**
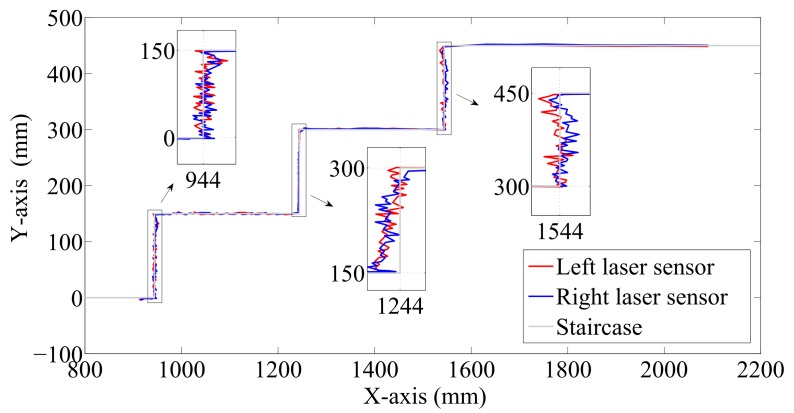
Estimated shape and size of the staircase.

The components of the distance measured can be calculated using expressions:

$$distance\left(x,y\right)=\left\{\begin{array}{cc} d_{sx} & =d_{s}\sin\left(k_{s}\right)\\ d_{sy} & =d_{s}\cos\left(k_{s}\right)-d_{so}\cos\left(k_{so}\right) \end{array}\right.$$

where $d_{s}$ is the distance measured by the laser sensors, $d_{sx}$ is the horizontal and $d_{sy}$ is the vertical component, $d_{so}$ is the initial measured distance and $k_{so}$ is the initial angle of the laser beams. The results calculated are illustrated in Figure 8.

The estimation of the stairs’ dimensions is achieved by partitioning the $d_{sx}$ and $d_{sy}$ measurements into groups, where a group is started whenever the distance between neighboring measurements exceeds a threshold of 50 mm. Figure 8 thus shows that there are three groups in the horizontal component (at approximately 944, 1244 and 1544 mm) and four in the vertical component (at approximately 0, 150, 300 and 450 mm). Finally, the median is calculated for each group, and the stairs’ dimensions and the number of stairs are therefore obtained.

**Figure 9:**
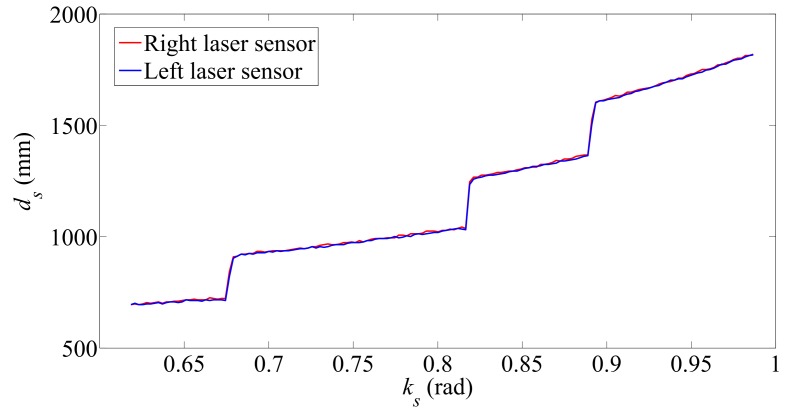
Distance measured during the scanning on the staircase surface before climbing down.

Another important advantage of our SCMS is its capacity to estimate the stairs’s dimensions when it goes down staircases. Figure 6b shows the scanning on the staircase surface before descent. Figure 9 shows the results obtained from the scanning of the staircase surface before climbing down. Note that in this case, the vertical part of the stairs cannot be scanned. Again, the overlapping among the resulting trajectories demonstrates that the SCMS are correctly aligned with the staircase.

**Figure 10:**
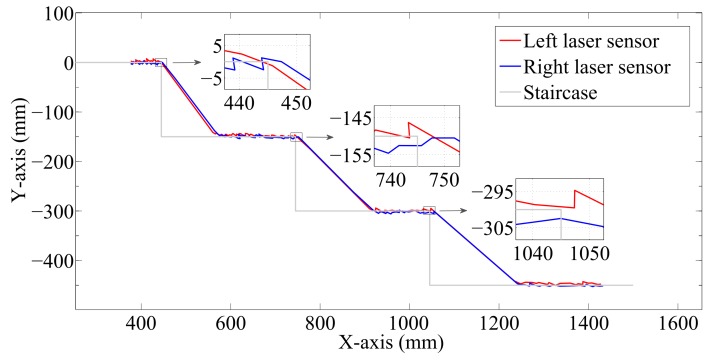
Estimated shape and size of the staircase.

The dimension estimation is achieved using the same approach as shown above. Figure 10 indicates that there are only four groups in the vertical component; at approximately 0, −150, −300 and −450 mm. The median is calculated for each group to obtain the height of the stairs and the number of steps. The estimation of the stairs’ width is based on the abrupt jumps of the laser beams, which occur three times. The right-hand ends of each group are calculated to determine the stairs width. The ends occur at proximity 445, 745 and 1045 mm.

The proposed strategy is easy to apply during both the ascent and descent of stairs. The estimation errors achieved are about 2–3 mm in both cases. These errors should not have a significant impact on the user’s safety and comfort. More details about the strategy used to estimate the size of the stairs are provided in [38].

## 3. Kinematics and Dynamics Modeling

The literature related to modeling SCMSs is currently limited, and one of the reasons for this is that these models are not required for a simple control law, which is in fact that most commonly adopted by electric powered wheelchairs. In the case of SCMSs, the definition of kinematic and dynamic models plays an important role not only in the design of control algorithms but also in the generation of optimal and smooth trajectories. This section is focused on the development of the kinematic and dynamic models for our hybrid SCMS, which takes into consideration its reconfigurable nature.

### 3.1. Kinematics Modeling

**Figure 11:**
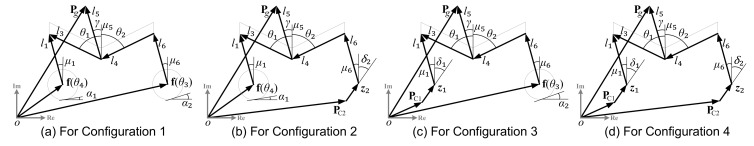
General kinematic scheme for the different configurations of the SCMS.

The kinematic model is used to define the relation among the position variables when the vehicle moves on either a continuous smooth profile or a discontinuous profile, such as a flat floor or a staircase. Figure 11 is very useful as regards understanding this relationship and formulating the kinematic equations for the four configurations that occur during stair-climbing. We shall begin by defining the notations used in the model and then determining the kinematic equations used in each configuration. The kinematic equations are expressed in complex notation, which has been chosen for mathematical and computational convenience.

The position of the center of mass of the SCMS is defined as $P_{g}={\left[P_{gx},P_{gy}\right]}^{T}$, where $P_{gx}$ and $P_{gy}$ are the horizontal and vertical components. The inclination angle of the SCMS frame is defined as γ. These values will be grouped into the vector $p={\left[P_{g}^{T},\gamma \right]}^{T}$. The front and rear joint variables of the positioning mechanism are denoted by $\theta_{1}$ and $\theta_{2}$, respectively. The positions of the front and rear axles of the wheels are represented using **f**($\theta_{4}$) and **f**($\theta_{3}$), where $\theta_{4}$ is the movement of the front wheels and $\theta_{3}$ is the movement of the rear wheels (the driving wheels). The positions of the front and rear sliding supports are denoted as $z_{1}$ and $z_{2}$. They form the angles $\delta_{1}$ and $\delta_{2}$ with the imaginary axis, respectively. $\theta_{1}$, $\theta_{2}$, $\theta_{3}$ and $\theta_{4}$ are rotational DOFs and $z_{1}$ and $z_{2}$ are translational DOFs. The actuated DOFs can be joined to the vector $q={\left[\theta_{1},\theta_{2},\theta_{3},z_{1},z_{2}\right]}^{T}$. The reference trajectories for the vectors p and q are defined as $p^{*}={\left[P_{g}^{*T},\gamma^{*}\right]}^{T}$ and $q^{*}={\left[\theta_{1}^{*},\theta_{2}^{*},\theta_{3}^{*},z_{1}^{*},z_{2}^{*}\right]}^{T}$, respectively.

Moreover, the components $P_{C1}$ and $P_{C2}$ correspond to the instant at which the wheels are off the ground during locomotion. The links that form a joint between the front climbing mechanism and the positioning mechanism are represented by the constants $l_{1}$ and $l_{3}$. The links that form a joint between the rear climbing mechanism and the positioning mechanism are denoted by the constants $l_{4}$ and $l_{6}$. The distance between the central joint and the position of the center of mass of the chassis ($P_{g}$) is, meanwhile, represented by $l_{5}$. The angles $\mu_{i}$ are defined to connect the vectors that comprise the general kinematic scheme. Each of the angles have a different constant value. In the kinematic model it is assumed that the rear and front wheels roll on both a flat surface ($\alpha_{i}=0$) and an uneven surface ($\alpha_{i}\neq 0$), and in both cases i=1,2. The direct kinematic model for each configuration is shown in Table 1. For a full description of the model, see [39,40,41].

Upon computing the difference between the two equations that define the current position of the SCMS in each of the four configurations and taking the imaginary part, an implicit expression (this implicit expression has a Jacobian form, which we call an implicit posture Jacobian model) that defines the posture of the SCMS can be obtained in a compact form as:

(5) $$F_{k}\left(q\right)=0$$

where, in this equation and those that follow, index *k* indicates the SCMS configuration (k=1,2,3or4).

In the kinematic model, it is assumed that the velocities of the active DOFs of the SCMS, $\dot{q}={\left[{\dot{\theta }}_{1},{\dot{\theta }}_{2},{\dot{\theta }}_{3},{\dot{z}}_{1},{\dot{z}}_{2}\right]}^{T}$, are the input control variables, and the derivative of the inclination angle of the SCMS frame, $\dot{\gamma }$, is the output. The differential relationship between the output γ and the variable q is therefore given by:

(6) $$\frac{\partial F_{k}}{\partial \gamma }\dot{\gamma }+\frac{\partial F_{k}}{\partial q}\dot{q}=0$$

The locomotion control is achieved by means of actuation on joints $\theta_{3}$, $z_{1}$ and $z_{2}$, while the posture control is achieved by means of actuation on joints $\theta_{1}$ and $\theta_{2}$. The relationship between the derivative of the controlled variable γ and the control signals ${\dot{\theta }}_{1}$ and ${\dot{\theta }}_{2}$ is therefore:

(7) $$\frac{\partial F_{k}}{\partial \gamma }\dot{\gamma }+\frac{\partial F_{k}}{\partial \theta_{1}}{\dot{\theta }}_{1}+\frac{\partial F_{k}}{\partial \theta_{1}}{\dot{\theta }}_{2}+g_{k}\left(q,\dot{q}\right)=0$$

in which:

$$g_{k}\left(q,\dot{q}\right)=\frac{\partial F_{k}}{\partial \theta_{3}}{\dot{\theta }}_{3}+\frac{\partial F_{k}}{\partial z_{1}}{\dot{z}}_{1}+\frac{\partial F_{k}}{\partial z_{2}}{\dot{z}}_{2}$$

The terms $\frac{\partial F_{k}}{\partial \gamma }$, $\frac{\partial F_{k}}{\partial \theta_{1}}$, $\frac{\partial F_{k}}{\partial \theta_{2}}$ and $g_{k}\left(q,\dot{q}\right)$ for the four configurations are shown in Table 2.

### 3.2. Dynamics Modeling

We develop a dynamic model under quasi-static conditions since the SCMS is assumed to be slow moving. The dynamic model is also different for each of the SCMS’s configurations and is expressed in terms of generalized coordinates. The relation between these coordinates and the system coordinates in each particular configuration is also different, and this difference is considered in order to adapt different equations to different configurations. We first obtain explicit expressions of the forces that appear in the mechanism in terms of the generalized coordinate variables, $r_{1}={\left[r_{1},r_{3}\right]}^{T}$ and $r_{2}={\left[r_{1},r_{4}\right]}^{T}$.

**Figure 12:**
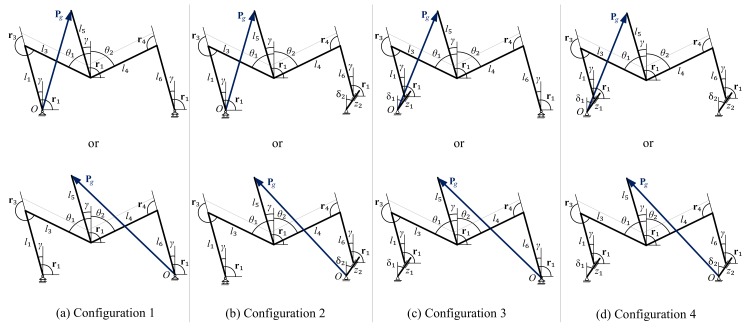
Definition of the generalized coordinate variables for each of the configurations of the SCMS.

Figure 12 shows the notation used to obtain the dynamics model for each of the SCMS’s configurations. With this notation, the position, velocity and acceleration of the center of mass can be written in the following general form:

(8) $${\bar{OP}}_{g}={\left[\begin{array}{c} {\bar{OP}}_{gx}\\ {\bar{OP}}_{gy} \end{array}\right]}_{k}={\left[\begin{array}{c} x_{1,2}\\ y_{1,2} \end{array}\right]}_{k},\;\;\;1\leq k\leq 4$$

(9) $${\dot{\bar{OP}}}_{g}={\left[\begin{array}{c} {\dot{\bar{OP}}}_{gx}\\ {\dot{\bar{OP}}}_{gy} \end{array}\right]}_{k}={\left[\begin{array}{c} {\dot{x}}_{1,2}\\ {\dot{y}}_{1,2} \end{array}\right]}_{k},\;\;\;1\leq k\leq 4$$

(10) $${\ddot{\bar{OP}}}_{g}={\left[\begin{array}{c} {\ddot{\bar{OP}}}_{gx}\\ {\ddot{\bar{OP}}}_{gy} \end{array}\right]}_{k}={\left[\begin{array}{c} {\ddot{x}}_{1,2}\\ {\ddot{y}}_{1,2} \end{array}\right]}_{k},\;\;\;1\leq k\leq 4$$

where subscripts 1 and 2 represent two ways in which to connect with the center of mass; subscript 1 is associated with $r_{1}$, which is generated on the front axle, and subscript 2 is associated with $r_{2}$, which is generated on the rear axle. The expressions ${\left[x_{1,2},y_{1,2}\right]}^{T}$, ${\left[{\dot{x}}_{1,2},{\dot{y}}_{1,2}\right]}^{T}$, ${\left[{\ddot{x}}_{1,2},{\ddot{y}}_{1,2}\right]}^{T}$ for Configuration 1 are shown as follows:

(11) $$\begin{array}{c} x_{1}=\left(l_{1}+l_{5}\right)C_{r_{1}}+l_{3}C_{\left(r_{1}+r_{3}\right)}\\ y_{1}=\left(l_{1}+l_{5}\right)S_{r_{1}}+l_{3}S_{\left(r_{1}+r_{3}\right)}\\ {\dot{x}}_{1}=-\left(l_{1}+l_{5}\right)S_{r_{1}}{\dot{r}}_{1}-l_{3}S_{\left(r_{1}+r_{3}\right)}\left({\dot{r}}_{1}+{\dot{r}}_{3}\right)\\ {\dot{y}}_{1}=\left(l_{1}+l_{5}\right)C_{r_{1}}{\dot{r}}_{1}+l_{3}C_{\left(r_{1}+r_{3}\right)}\left({\dot{r}}_{1}+{\dot{r}}_{3}\right)\\ {\ddot{x}}_{1}=-\left(l_{1}+l_{5}\right)\left(C_{r_{1}}{\dot{r}}_{1}^{2}+S_{r_{1}}{\ddot{r}}_{1}\right)-l_{3}\left[C_{\left(r_{1}+r_{3}\right)}{\left({\dot{r}}_{1}+{\dot{r}}_{3}\right)}^{2}+S_{\left(r_{1}+r_{3}\right)}\left({\ddot{r}}_{1}+{\ddot{r}}_{3}\right)\right]\\ {\ddot{y}}_{1}=\left(l_{1}+l_{5}\right)\left(-S_{r_{1}}{\dot{r}}_{1}^{2}+C_{r_{1}}{\ddot{r}}_{1}\right)+l_{3}\left[-S_{\left(r_{1}+r_{3}\right)}{\left({\dot{r}}_{1}+{\dot{r}}_{3}\right)}^{2}+C_{\left(r_{1}+r_{3}\right)}\left({\ddot{r}}_{1}+{\ddot{r}}_{3}\right)\right] \end{array}$$

or:

(12) $$\begin{array}{c} x_{2}=\left(l_{6}+l_{5}\right)C_{r_{1}}+l_{4}C_{\left(r_{1}+r_{4}\right)}\\ y_{2}=\left(l_{6}+l_{5}\right)S_{r_{1}}+l_{4}S_{\left(r_{1}+r_{4}\right)}\\ {\dot{x}}_{2}=-\left(l_{6}+l_{5}\right)S_{r_{1}}{\dot{r}}_{1}-l_{4}S_{\left(r_{1}+r_{4}\right)}\left({\dot{r}}_{1}+{\dot{r}}_{4}\right)\\ {\dot{y}}_{2}=\left(l_{6}+l_{5}\right)C_{r_{1}}{\dot{r}}_{1}+l_{4}C_{\left(r_{1}+r_{4}\right)}\left({\dot{r}}_{1}+{\dot{r}}_{4}\right)\\ {\ddot{x}}_{2}=-\left(l_{6}+l_{5}\right)\left[C_{r_{1}}{\dot{r}}_{1}^{2}+S_{r_{1}}{\ddot{r}}_{1}\right]-l_{4}\left[C_{\left(r_{1}+r_{4}\right)}{\left({\dot{r}}_{1}+{\dot{r}}_{4}\right)}^{2}+S_{\left(r_{1}+r_{4}\right)}\left({\ddot{r}}_{1}+{\ddot{r}}_{4}\right)\right]\\ {\ddot{y}}_{2}=\left(l_{6}+l_{5}\right)\left[-S_{r_{1}}{\dot{r}}_{1}^{2}+C_{r_{1}}{\ddot{r}}_{1}\right]+l_{4}\left[-S_{\left(r_{1}+r_{4}\right)}{\left({\dot{r}}_{1}+{\dot{r}}_{4}\right)}^{2}+C_{\left(r_{1}+r_{4}\right)}\left({\ddot{r}}_{1}+{\ddot{r}}_{4}\right)\right] \end{array}$$

In these equations and those that follow, $\sin\left(\cdot \right)\equiv S_{\left(\cdot \right)}$ and $\cos\left(\cdot \right)\equiv C_{\left(\cdot \right)}$.

Let $F_{m}$ and $F_{g}$ be the vectors of forces acting on the center of mass of the SCMS ($P_{g}$), generated as a result of the acceleration of the system and gravity, respectively. $F_{m}$ and $F_{g}$ can be obtained as follows:

(13) $$F_{m}=m\cdot {\ddot{\bar{OP}}}_{g}=m\cdot {\left[{\ddot{x}}_{1,2}\;{\ddot{y}}_{1,2}\right]}^{T},$$

(14) $$\;F_{g}=m{\left[0\;-g\right]}^{T},$$

where *m* is the mass of the whole system (SCMS + passenger). It is important to mention two considerations regarding the dynamic model: first, that the mass of the system is invariant; and second, that the position of the center of mass barely changes during the stair-climbing process (it is assumed that the user will not make abrupt movements [42,43]).

If the results of the forces provided by Expressions (13) and (14) are grouped, and Newton’s Second Law is applied, the generalized forces exerted on the center of mass of the mechanism, $F_{P_{g}}$, are obtained from the following expressions:

(15) $$F_{P_{g}}=m\cdot \left[\begin{array}{c} A_{1}\cdot \left[\begin{array}{c} {\ddot{r}}_{1}\\ {\ddot{r}}_{3} \end{array}\right]+B_{1}\cdot \left[\begin{array}{c} {\dot{r}}_{1}\\ {\dot{r}}_{3} \end{array}\right]+\left[\begin{array}{c} 0\\ -g \end{array}\right] \end{array}\right]$$

where:

$$A_{1}=\left[\begin{array}{cc} -\left(l_{1}+l_{5}\right)S_{r_{1}}-l_{3}S_{\left(r_{1}+r_{3}\right)} & -l_{3}S_{\left(r_{1}+r_{3}\right)}\\ \left(l_{1}+l_{5}\right)C_{r_{1}}+l_{3}C_{\left(r_{1}+r_{3}\right)} & l_{3}C_{\left(r_{1}+r_{3}\right)} \end{array}\right]$$

$$B_{1}=\left[\begin{array}{cc} -\left[\left(l_{1}+l_{5}\right)C_{r_{1}}+l_{3}C_{\left(r_{1}+r_{3}\right)}\right]{\dot{r}}_{1} & -l_{3}C_{\left(r_{1}+r_{3}\right)}\left(2{\dot{r}}_{1}+{\dot{r}}_{3}\right)\\ -\left[\left(l_{1}+l_{5}\right)S_{r_{1}}+l_{3}S_{\left(r_{1}+r_{3}\right)}\right]{\dot{r}}_{1} & -l_{3}S_{\left(r_{1}+r_{3}\right)}\left(2{\dot{r}}_{1}+{\dot{r}}_{3}\right) \end{array}\right]$$

or:

(16) $$F_{P_{g}}=m\cdot \left[\begin{array}{c} A_{2}\cdot \left[\begin{array}{c} {\ddot{r}}_{1}\\ {\ddot{r}}_{4} \end{array}\right]+B_{2}\cdot \left[\begin{array}{c} {\dot{r}}_{1}\\ {\dot{r}}_{4} \end{array}\right]+\left[\begin{array}{c} 0\\ -g \end{array}\right] \end{array}\right]$$

where:

$$A_{2}=\left[\begin{array}{cc} -\left(l_{6}+l_{5}\right)S_{r_{1}}-l_{4}S_{\left(r_{1}+r_{4}\right)} & -l_{4}S_{\left(r_{1}+r_{4}\right)}\\ \left(l_{6}+l_{5}\right)C_{r_{1}}+l_{4}C_{\left(r_{1}+r_{4}\right)} & l_{4}C_{\left(r_{1}+r_{4}\right)} \end{array}\right]$$

$$B_{2}=\left[\begin{array}{cc} -\left[\left(l_{6}+l_{5}\right)C_{r_{1}}+l_{4}C_{\left(r_{1}+r_{4}\right)}\right]{\dot{r}}_{1} & -l_{4}C_{\left(r_{1}+r_{4}\right)}\left(2{\dot{r}}_{1}+{\dot{r}}_{4}\right)\\ -\left[\left(l_{6}+l_{5}\right)S_{r_{1}}+l_{4}S_{\left(r_{1}+r_{4}\right)}\right]{\dot{r}}_{1} & -l_{4}S_{\left(r_{1}+r_{4}\right)}\left(2{\dot{r}}_{1}+{\dot{r}}_{4}\right) \end{array}\right]$$

Moreover, it is well known that under quasi-static conditions, the relationship between the torques exerted on the joints and the forces and torques exerted on the center of gravity of the mechanism are related by mean of the following expression:

(17) $$\tau =J^{T}\left(r_{1,2}\right)F_{P_{g}},$$

in which the Jacobians, $J(r_{1})$ and $J(r_{2})$, are obtained directly from Equation (8) as follows:

(18) $$J\left(r_{1}\right)={\left[\begin{array}{cc} \frac{\partial x}{\partial r_{1}} & \frac{\partial x}{\partial r_{3}}\;\\ \frac{\partial y}{\partial r_{1}} & \frac{\partial y}{\partial r_{3}} \end{array}\right]}_{k}\;\;\;1\leq k\leq 4$$

or:

(19) $$J\left(r_{2}\right)={\left[\begin{array}{cc} \frac{\partial x}{\partial r_{1}} & \frac{\partial x}{\partial r_{4}}\;\\ \frac{\partial y}{\partial r_{1}} & \frac{\partial y}{\partial r_{4}} \end{array}\right]}_{k}\;\;\;1\leq k\leq 4$$

and the values of the partial derivatives are provided by the following expressions:

(20) $$J\left(r_{1}\right)=\left[\begin{array}{cc} -\left(l_{1}+l_{5}\right)S_{r_{1}}-l_{3}S_{\left(r_{1}+r_{3}\right)} & -l_{3}S_{\left(r_{1}+r_{3}\right)}\\ \left(l_{1}+l_{5}\right)C_{r_{1}}+l_{3}C_{\left(r_{1}+r_{3}\right)} & l_{3}C_{\left(r_{1}+r_{3}\right)} \end{array}\right]$$

or

(21) $$J\left(r_{1}\right)=\left[\begin{array}{cc} -\left(l_{6}+l_{5}\right)S_{r_{1}}-l_{4}S_{\left(r_{1}+r_{4}\right)} & -l_{4}S_{\left(r_{1}+r_{4}\right)}\\ \left(l_{6}+l_{5}\right)C_{r_{1}}+l_{4}C_{\left(r_{1}+r_{4}\right)} & l_{4}C_{\left(r_{1}+r_{4}\right)} \end{array}\right]$$

Upon substituting Equations (20) and (21) in Equation (17), and after certain algebraic manipulations, the following dynamics model is obtained for the SCMS:

(22) $$\;\tau =B\left(r\right)\ddot{r}+C\left(r,\;\dot{r}\right)\dot{r}+G\left(r\right),$$

where the values of the matrices B(r), $C(r,\;\dot{r})$ and G(r) are provided by the following expressions:

$$\begin{array}{l} B(r_{1})=m\left[\begin{array}{cc} {\left(l_{1}+l_{5}\right)}^{2}+l_{3}^{2}+2l_{3}\left(l_{1}+l_{5}\right)C_{r_{3}} & l_{3}^{2}+l_{3}\left(l_{1}+l_{5}\right)C_{r_{3}}\\ l_{3}^{2}+l_{3}\left(l_{1}+l_{5}\right)C_{r_{3}} & l_{3}^{2} \end{array}\right]\\ C(r_{1},\;\dot{r_{1}})=m\left[\begin{array}{cc} 0 & -l_{3}\left(l_{1}+l_{5}\right)S_{r_{3}}\left(2{\dot{r}}_{1}+{\dot{r}}_{3}\right)\\ l_{3}\left(l_{1}+l_{5}\right)S_{r_{3}}{\dot{r}}_{1} & 0 \end{array}\right]\\ G(r_{1})=mg\left[\begin{array}{c} \left(l_{1}+l_{5}\right)C_{r_{1}}+l_{3}C_{\left(r_{1}+r_{3}\right)}\\ l_{3}C_{\left(r_{1}+r_{3}\right)} \end{array}\right] \end{array}$$

or:

$$\begin{array}{l} B(r_{2})=m\left[\begin{array}{cc} {\left(l_{6}+l_{5}\right)}^{2}+l_{4}^{2}+2l_{4}\left(l_{6}+l_{5}\right)C_{r_{4}} & l_{4}^{2}+l_{4}\left(l_{6}+l_{5}\right)C_{r_{4}}\\ l_{4}^{2}+l_{4}\left(l_{6}+l_{5}\right)C_{r_{4}} & l_{4}^{2} \end{array}\right]\\ C(r_{2},\;{\dot{r}}_{2})=m\left[\begin{array}{cc} 0 & -l_{4}\left(l_{6}+l_{5}\right)S_{r_{4}}\left(2{\dot{r}}_{1}+{\dot{r}}_{4}\right)\\ l_{4}\left(l_{6}+l_{5}\right)S_{r_{4}}{\dot{r}}_{1} & 0 \end{array}\right]\\ G(r_{2})=mg\left[\begin{array}{c} \left(l_{6}+l_{5}\right)C_{r_{1}}+l_{4}C_{\left(r_{1}+r_{4}\right)}\\ l_{4}C_{\left(r_{1}+r_{4}\right)} \end{array}\right] \end{array}$$

The computation of articular variables and their corresponding derivatives ($r_{1}=[r_{1},r_{3}{]}^{T},r_{2}=[r_{1},r_{4}{]}^{T}$, ${\dot{r}}_{1}=[{\dot{r}}_{1},{\dot{r}}_{3}{]}^{T},\;{\dot{r}}_{2}=[{\dot{r}}_{1},{\dot{r}}_{4}{]}^{T}$, ${\ddot{r}}_{1}=[{\ddot{r}}_{1},{\ddot{r}}_{3}{]}^{T}\mathrm{and}\;{\ddot{r}}_{2}=[{\ddot{r}}_{1},{\ddot{r}}_{4}{]}^{T}$), which are expressed in terms of the system variables (q = [$\theta_{1},\theta_{2},\theta_{3},z_{1},z_{2}{]}^{T}$, $\dot{q}$ = [${\dot{\theta }}_{1},{\dot{\theta }}_{2},{\dot{\theta }}_{3},{\dot{z}}_{1},{\dot{z}}_{2}{]}^{T}$ and $\ddot{q}$ = [${\ddot{\theta }}_{1},{\ddot{\theta }}_{2},{\ddot{\theta }}_{3},{\ddot{z}}_{1},{\ddot{z}}_{2}{]}^{T}$), will depend on the particular configuration of the mechanism. It is, therefore, necessary to find a relationship between the articular variables and the system variables for all the possible configurations and to use the model provided by Equation (22) in order to obtain a new model as a function of **q** rather than **r**, i.e.,

(19) $$\;\tau =B\left(q\right)\ddot{q}+C\left(q,\;\dot{q}\right)\dot{q}+G\left(q\right),$$

If the proposed dynamics model and the system geometry are used and we take advantage of the fact that the front and rear frames of the positioning mechanism do not rotate with regard to the central frame, the following relationships are obtained (see Figure 12):

(24) $$r_{1}=\frac{\pi }{2}+\gamma ;\;\;\;r_{3}=\pi +\theta_{1};\;\;\;r_{4}=\pi -\theta_{2}$$

The relationships for velocities and accelerations are obtained by differentiating the expressions shown above:

(25) $${\dot{r}}_{1}=\dot{\gamma };\;\;\;{\dot{r}}_{3}={\dot{\theta }}_{1};\;\;\;{\dot{r}}_{4}=-{\dot{\theta }}_{2}$$

(26) $${\ddot{r}}_{1}=\ddot{\gamma };\;\;\;{\ddot{r}}_{3}={\ddot{\theta }}_{1};\;\;\;{\ddot{r}}_{4}=-{\ddot{\theta }}_{2}$$

This system property makes it necessary to obtain mathematical relationships between the articular variables and control variables of the actuated DOF of the SCMS for all possible configurations. As is shown in Figure 12, the vertical component of the center of mass is the same in both cases in each of the configurations. We can therefore assume that:

(27) $${\bar{OP}}_{g_{y_{1}}}={\bar{OP}}_{g_{y_{2}}}$$

(28) $${\dot{\bar{OP}}}_{g_{y_{1}}}={\dot{\bar{OP}}}_{g_{y_{2}}}$$

(29) $${\ddot{\bar{OP}}}_{g_{y_{1}}}={\ddot{\bar{OP}}}_{g_{y_{2}}}$$

Upon taking the aforementioned considerations into account, substituting relations (24)–(26) in Equations (11) and (12), and after certain algebraic manipulations, the first and second derivatives of the inclination angle of the SCMS frame with regard to time ($\dot{\gamma }$ and $\ddot{\gamma }$) are obtained:

(30) $$\dot{\gamma }=\frac{-l_{3}S_{\left(\gamma +\theta_{1}\right)}{\dot{\theta }}_{1}-l_{4}S_{\left(\gamma -\theta_{2}\right)}{\dot{\theta }}_{2}}{\left(l_{1}-l_{6}\right)S_{\left(\gamma \right)}+l_{4}S_{\left(\gamma -\theta_{2}\right)}-l_{3}S_{\left(\gamma +\theta_{1}\right)}}\;$$

(31) $$\begin{array}{c} \ddot{\gamma }=\frac{\left(l_{1}-l_{6}\right)C_{\left(\gamma \right)}{\dot{\gamma }}^{2}+l_{3}\left[C_{\left(\gamma +\theta_{1}\right)}{\left(\dot{\gamma }+{\dot{\theta }}_{1}\right)}^{2}-S_{\left(\gamma +\theta_{1}\right)}{\ddot{\theta }}_{1}\right]-l_{4}\left[C_{\left(\gamma -\theta_{2}\right)}{\left(\dot{\gamma }-{\dot{\theta }}_{2}\right)}^{2}+S_{\left(\gamma -\theta_{2}\right)}{\ddot{\theta }}_{2}\right]}{\left(l_{1}-l_{6}\right)S_{\left(\gamma \right)}+l_{4}S_{\left(\gamma -\theta_{2}\right)}-l_{3}S_{\left(\gamma +\theta_{1}\right)}} \end{array}$$

## 4. Definition of the Trajectory Generator

During the stair-climbing process, the SCMS must realize a trajectory from an initial state to a final one, both of which are characterized by null velocities and null accelerations (this occurs every time that the SCMS changes from one configuration to another). Solving the optimal trajectory planning problem involves the determination of the transfer time *T*, the trajectory $ℑ\left(t\right)=(P_{gx}\left(t\right),P_{gy}\left(t\right),\gamma \left(t\right))$ and the corresponding input controls $\Gamma \left(t\right)=(\theta_{1}\left(t\right),\theta_{2}\left(t\right),\theta_{3}\left(t\right),z_{1}\left(t\right),z_{2}\left(t\right))$ such that the initial and final states are matched, constraints are respected and a cost function is minimized. Hereafter, we exclusively adopt the following cost function:

(31) $$F^{obj}=\alpha \int_{0}^{T}dt+\left(1-\alpha \right)\;\frac{1}{T}\int_{0}^{T}\gamma^{2}\left(t\right)dt$$

where 0≤α≤1. This function provides a compromise between the stair-climbing time process and the passenger’s comfort, thus allowing the threshold of accuracy to be selected according to the specific needs of application.

The boundary conditions of position, velocity and acceleration inherent to the achievement of the optimal trajectory planning are those imposed on the SCMS:

(33) $$ℑ\left(0\right)={ℑ}_{ini}\;\;\mathrm{and}\;\;ℑ\left(T\right)={ℑ}_{fin}$$

(34) $$\dot{ℑ}\left(0\right)=\vec{0}\;\;\mathrm{and}\;\;\dot{ℑ}\left(T\right)=\vec{0}$$

(35) $$\ddot{ℑ}\left(0\right)=\vec{0}\;\;\mathrm{and}\;\;\ddot{ℑ}\left(T\right)=\vec{0}$$

Other boundary conditions that need to be fulfilled during the stair-climbing movement are provided below:

- A function Tran(ℑ(t)) is used to indicate whether it is possible to change the configuration of the SCMS:
  (36) Tran(ℑ(t))=true
- Bounds on the SCMS configurations:
  (37) $${ℑ}_{min}\leq ℑ\left(t\right)\leq {ℑ}_{max}$$
  These bounds make it possible for the SCMS to surpass stairs of different sizes and to maintain an inclination with regard to the direction of gravity of its chassis (γ=0).
- Bounds on the actuator velocities and accelerations:
  (38) $${\dot{\theta_{i}}}_{min}\leq \dot{\theta_{i}}\left(t\right)\leq {\dot{\theta_{i}}}_{max},\;\;\;i=1,2\;\mathrm{and}\;3$$
  (39) $${\ddot{\theta_{i}}}_{min}\leq \ddot{\theta_{i}}\left(t\right)\leq {\ddot{\theta_{i}}}_{max},\;\;\;i=1,2\;\mathrm{and}\;3$$
  (40) $${\dot{z_{i}}}_{min}\leq \dot{z_{i}}\left(t\right)\leq {\dot{z_{i}}}_{max},\;\;\;i=1\;\mathrm{and}\;2$$
  (41) $${\ddot{z_{i}}}_{min}\leq \ddot{z_{i}}\left(t\right)\leq {\ddot{z_{i}}}_{max},\;\;\;i=1\;\mathrm{and}\;2$$
  which arise from the fact that each motor or actuator has a limited speed of operation. These bounds ensure that the inertial forces are smaller than the gravitational force. The mass center accelerations and velocities of the whole system (SCMS + passenger) must therefore be less than the maximum comfort acceleration and velocity, respectively. The inclination angle must have the acceptable comfort ranges, ±10${}^{\circ }$.
- Bounds on the comfort velocities and accelerations:
  (42) $${\dot{\gamma }}_{min}\leq \dot{\gamma }\left(t\right)\leq {\dot{\gamma }}_{max}$$
  (43) $${\ddot{\gamma }}_{min}\leq \ddot{\gamma }\left(t\right)\leq {\ddot{\gamma }}_{max}$$
- A function Col(ℑ(t)) is also used to indicate whether or not a given wheel or sliding support of the SMCS is accidentally colliding with an obstacle (Information regarding the environment is obtained by using the strategy designed to estimate the size and shape of the stairs, which was presented in Section 2.3. In this case, knowledge about the environment allows the proposed prototype to determine whether or not a collision will occur):
  (44) Col(ℑ(t))=false

It is possible to use Equations (30) and (31) to express all the elements of the optimization problem (cost function and constraints) as a function of the time evolution of only five configuration parameters, namely {$\theta_{1}\left(t\right),\theta_{2}\left(t\right),\theta_{3}\left(t\right),z_{1}\left(t\right),z_{2}\left(t\right)$} and their time derivatives. In fact, it is possible to define a trajectory candidate by defining the evolution of {$\theta_{1}\left(t\right),\theta_{2}\left(t\right),\theta_{3}\left(t\right),z_{1}\left(t\right),z_{2}\left(t\right)$}, for t∈[0,T], while accounting for boundary conditions (33)–(35) and bounds (36)–(44) in the SCMS configurations.

The remaining question is how to generate the trajectory candidates. We therefore propose considering that any trajectory candidate is a composition of: (i) a parametric form $P\left(\upsilon \right)=(\theta_{1}\left(\upsilon \right),\theta_{2}\left(\upsilon \right),\theta_{3}\left(\upsilon \right),z_{1}\left(\upsilon \right),z_{2}\left(\upsilon \right))$, υ∈[0,1], which defines the SCMS path; and (ii) a monotonically increasing function υ(t), t∈[0,T], which specifies the motion on this path, i.e.,

(45) ℑ(t)=P(υ)∘υ(t)

**Figure 13:**
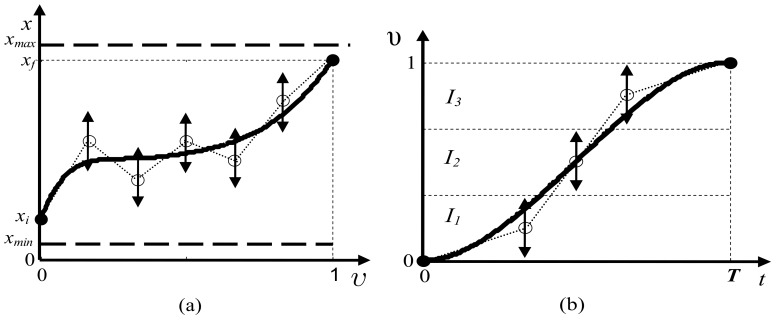
Path and motion profiles. (**a**) A path component profile and (**b**) the motion profile.

The optimal evolutions of P(υ) and υ(t), which are approximated using B-spline functions by fitting a set of control points, are found using a nonlinear optimization technique. It is possible to construct P(υ) and υ(t) as follows:

- Generate P(υ), υ∈[0,1] (Figure 13a) by means of a quintic B-spline model. The fifth degree ensures the continuity of the second order derivatives of angles $\theta_{1},\theta_{2},\theta_{3},z_{1},$ and $z_{2}$. This B-spline is built by using a set of $N_{p}$ control points (five points at least), and is generated within the admissible workspace defined by (37) and by accounting for constraints (33) and (44).
- Build the motion profile υ(t) on the interval [0,T] using a quintic B-spline model (Figure 13b), generated by $N_{m}$ control points that are uniformly distributed throughout the time scale, and by accounting for the following boundary conditions:
  $$\upsilon \left(0\right)=0\;\;\dot{\upsilon }\left(0\right)=0\;\;\ddot{\upsilon }\left(0\right)=0\;\;\dddot{\upsilon }\left(0\right)=0$$
  $$\upsilon \left(T\right)=1\;\;\dot{\upsilon }\left(T\right)=0\;\;\ddot{\upsilon }\left(T\right)=0\;\;\dddot{\upsilon }\left(T\right)=0\;$$

These conditions ensure the compatibility of the resulting trajectory ℑ(t) with constraints (34) and (35). The increasing monotony of the motion profile is ensured by generating the $N_{m}$ control points in $I_{N_{m}}$ intervals, as shown in (Figure 13b):

$$I_{i}=\left[\frac{i-1}{N_{m}},\frac{i}{N_{m}}\right]\;\;\;i=1,\ldots ,\;N_{m}$$

The design parameters of P(υ) and υ(t), which are the coordinates of the B-spline function control points and the value of *T*, become the sole unknown values of the trajectory generation problem.

### Optimization and the SCMS’s Control Scheme

**Figure 14:**
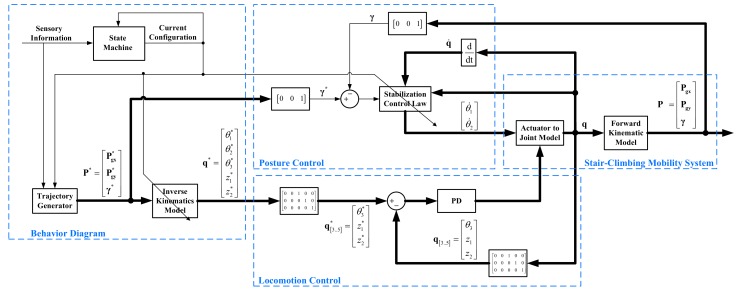
Control scheme of the proposed SCMS.

The control scheme shown in Figure 14 was designed and implemented in order to achieve an accurate tracking of the desired inclination angle ($\gamma^{*}=0$) and the desired center of mass trajectory ($P_{g}^{*}$) during the stair-climbing process. In this scheme, the trajectory generator of the behavior diagram module was designed without taking trajectory optimization into account.

**Figure 15:**
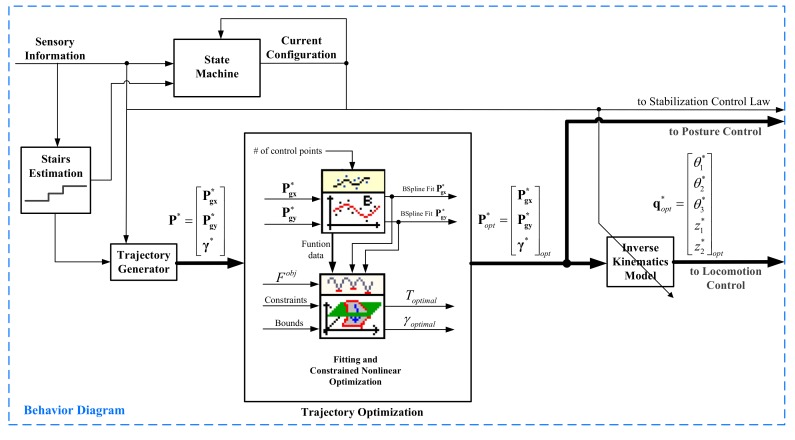
Scheme of the optimal trajectory generator based on constrained nonlinear optimization.

In order to solve the trajectory optimization problem proposed in this paper, a sub-module, called trajectory optimization, has been incorporated into the behavior diagram module, as shown in Figure 15.

In the modified module, the State Machine controls the sequence of states that the SCMS should pass through. The trajectory generator provides the desired trajectory profiles $P^{*}$ that the SCMS will track. Both the machine and the generator depend directly on the information provided by the sensory system and on the previous and current configurations. The generator also depends on the knowledge of the dimensions of the stairs. In the trajectory optimization sub-module, the reference trajectories $P^{*}$ are then optimized by using the approach shown in Figure 15. In this sub-module, the reference trajectories $P_{opt}^{*}$ are then generated and these are used to control the system.

## 5. Experimental Results

This section provides details of the experimental setup used to evaluate the development of the hybrid SCMS using the trajectory optimization proposed in this paper.

### 5.1. Experimental Setup

**Figure 16:**
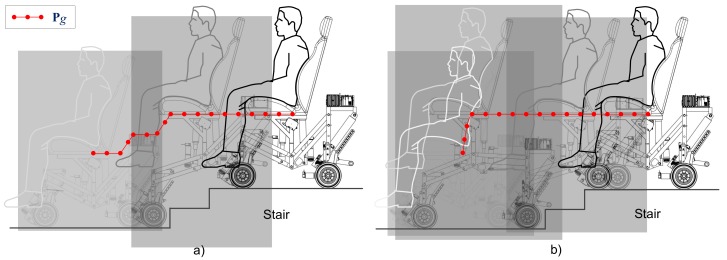
Position profiles of the center of mass of the SCMS used in the (**a**) first and (**b**) second experiments.

Two stair-climbing experiments were configured in order to make the SCMS climb up a two-step staircase. In both experiments, the SCMS was controlled using the control scheme shown in Figure 14 in conjunction with a PI controller proposed in [44]. The position profile of the center of mass ($P_{g}$) used in the first experiment consisted of straight lines with slopes of 0 and 5° (see Figure 16a). The horizontal lines are attributed to Configurations 1 and 3 while the others are attributed to Configuration 2. This approach has two advantages: the control is simplified and the power consumption is minimized. The position profile used in the second experiment consisted of two straight lines, one vertical and the other horizontal (see Figure 16b). The vertical line is generated at the beginning of the stair-climbing process (with Configuration 1), while the horizontal line is generated in the rest of the process (with all the configurations). The main advantage of this approach is observed during locomotion, during which the effect of climbing stairs is minimized. Both profiles have been generated by considering the information regarding stair geometry.

In the experiments, a volunteer was seated on the hybrid SCMS in order to obtain a real interaction between the user and the system. The experiments were performed using the parameters shown in Table 3, Table 4 and Table 5.

**Figure 17:**
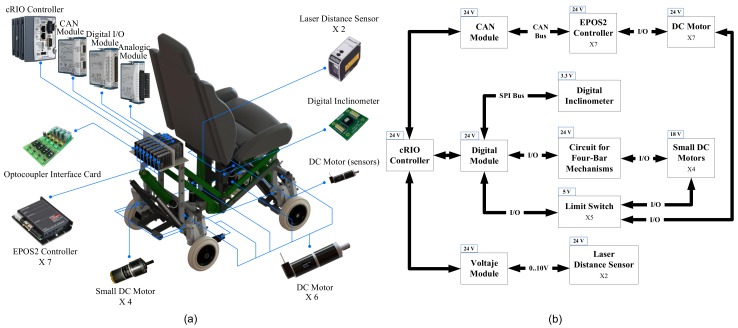
(**a**) Physical and (**b**) functional perspective of the hardware control system.

The control system of the proposed SCMS incorporates a reliable and robust hardware that has been chosen by taking into account the technical aspects of control and communication. The physical hardware of the control system and its general architecture are illustrated in Figure 17.

The control system architecture and the interface for the communication system have been developed for the purpose of solving the stair-climbing problem in an efficient and autonomous manner that ensures the passenger’s safety and comfort. The proposed SCMS is controlled by an advanced reconfigurable control and acquisition system, CompactRIO, which is manufactured by National Instruments (NI). It consists of a real-time processor that is connected to a reconfigurable chassis using a local Peripheral Component Interconnect (PCI) bus interface. The overall control has been developed using the CompactRIO system with LabVIEW based control programs. The Qualisys Optotrack motion capture system was also used to capture the position of the center of mass ($P_{g}$).

Functions such as “Quadratic Programming.vi”, “B-Spline Fit.vi” and “Constrained Nonlinear Optimization.vi” are software units coded in LabVIEW and copyrighted from National Instruments used for testing when solving the optimization problems, along with other programming tools and solvers of differential equations. In this work, the optimization task is performed by using both the “B-Spline Fit” and the “Constrained Nonlinear Optimization” Virtual Instruments (VIs). The documentation of the functions is referred to in [45,46], respectively.

**Figure 18:**
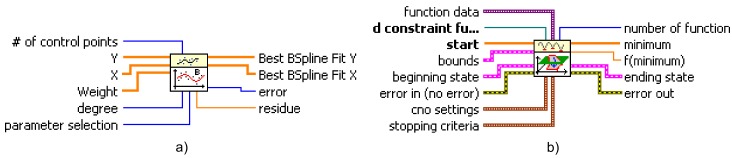
The two main Virtual Instruments (VIs) used to solve the optimization problem; (**a**) the B-Spline Fit VI and (**b**) the Constrained Nonlinear Optimization VI.

The Virtual Instrument (VI) (LabVIEW is a graphical programming language that uses icons rather than lines of text to create applications. LabVIEW programs are called Virtual Instruments (VIs) because their appearance and operation imitate physical instruments, such as generators, oscilloscopes, analyzers and multimeters) “B-Spline Fit” (see Figure 18a), one of the LabVIEW fitting routines, is used to approximate P(υ) and υ(t). Their optimal values are approximated by fitting a set of control points that are easily found using an SQP technique. In these experiments, P(υ) and υ(t) are generated by means of a quintic B-spline model. The fifth degree ensures the continuity of the second derivative of the paths. In P(υ), the B-spline is built by using a set of five control points, while in υ(t), it is generated by $N_{m}$ control points that are uniformly distributed throughout the time scale. Both B-splines are generated within the admissible workspace and by accounting for constraints and boundary conditions. Solutions based on SQP techniques have been proposed to generate collision-free smooth paths for unmanned aerial vehicles (see, for example, [47,48,49]), autonomous ground vehicles (see, for example, [50,51,52,53]) and electric powered wheelchairs (see, for example, [54,55,56,57,58]). This is, however, to the best of our knowledge, the first time that this sort of trajectory generator has been developed for systems of a reconfigurable nature (discontinuous states) and applied to surpass non-continuous architectural barriers (stairs).

The VI “Constrained Nonlinear Optimization” (see Figure 18b), one of the LabVIEW optimization routines is, meanwhile, used to solve the optimization problem (cost function and constraints). This VI solves a general nonlinear optimization problem with nonlinear equality and nonlinear inequality constraint bounds, also using an SQP method. This function implements the SQP algorithm in a generic form. The function expects a continuous-time formulation of the objective function and constraints, limits on the decision variables and initial estimation values, as regards both the decision variables and the state of the algorithm. Besides the SQP algorithm, confronting the formulation of the nonlinear optimization problem involves solving the set of equations in the model, and evaluating the solution. This numerical approach is widely used when direct analytical estimates cannot be determined [59,60,61].

**Figure 19:**
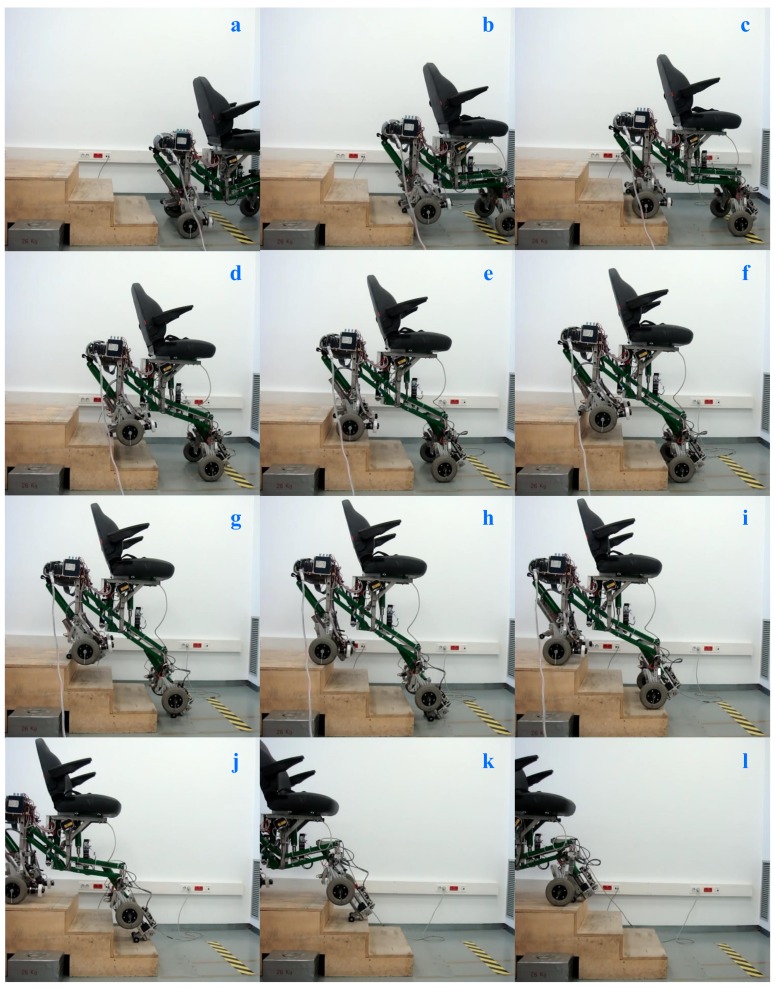
Sequence of climbing process when it surpasses a three-step staircase. All possible configurations of the SCMS are included; Configuration 1 in (**a**,**c**,**e**,**i**,**l**); Configuration 2 in (**b**,**d**,**f**); Configuration 3 in (**h**,**j**,**k**); and Configuration 4 in (**g**). See the video referenced in the Supplementary Materials.

**Figure 20:**
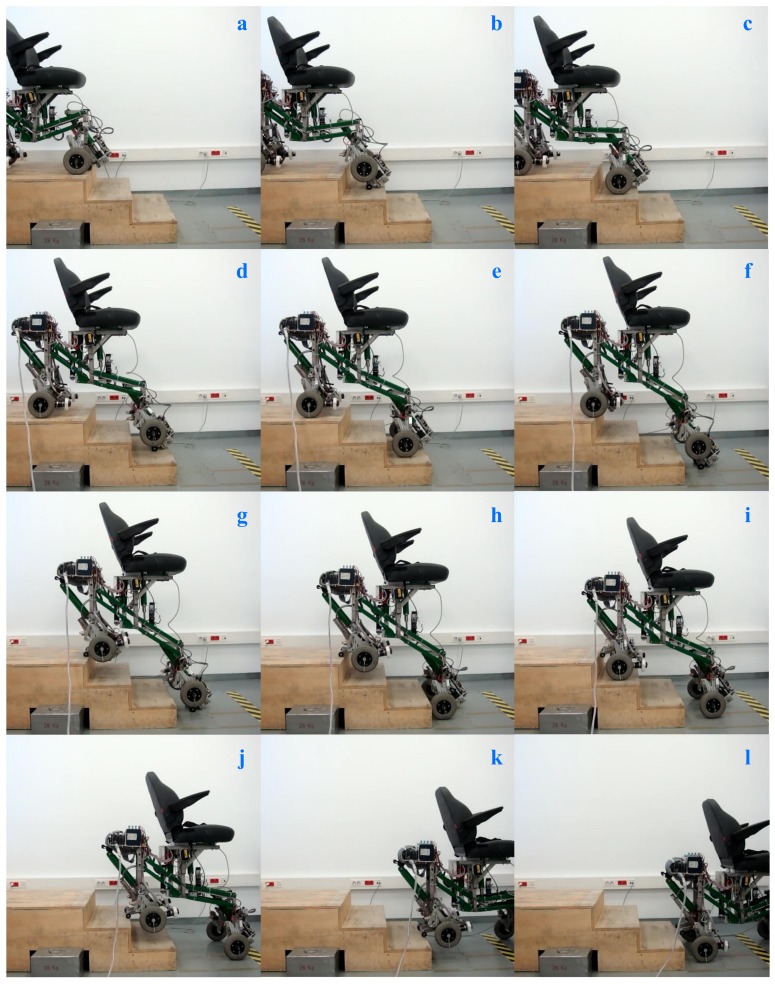
Sequence of descent process when it surpasses a three-step staircase. All possible configurations of the SCMS are included; Configuration 1 in (**a**,**c**,**e**,**i**,**l**); Configuration 2 in (**h**,**j**,**k**); Configuration 3 in (**b**,**d**,**f**); and Configuration 4 in (**g**). See the video referenced in the Supplementary Materials.

Finally, visual sequences (and Videos S1 and S2 referenced in the Supplementary Materials) that exhibit the climbing and descending process of a three-step staircase are illustrated in Figure 19 and Figure 20. We would like to mention that videos associated with descending stairs are not usually shown because in these types of tests the non-commercial experimental prototypes are susceptible to having stability problems and therefore a greater risk of falls. In the case of our experimental prototype, this problem is completely solved because it always transits between mechanically stable configurations. Our prototype is characterized by the fact that it maintain a stable equilibrium on the stair when it changes from one configuration to another.

### 5.2. Results

The results of the experiments are presented in two parts; the first shows the results of the first experiment (Stair-Climbing Mode 1), while the second depicts the results of the second experiment (Stair-Climbing Mode 2).

#### 5.2.1. First Experiment

**Figure 21:**
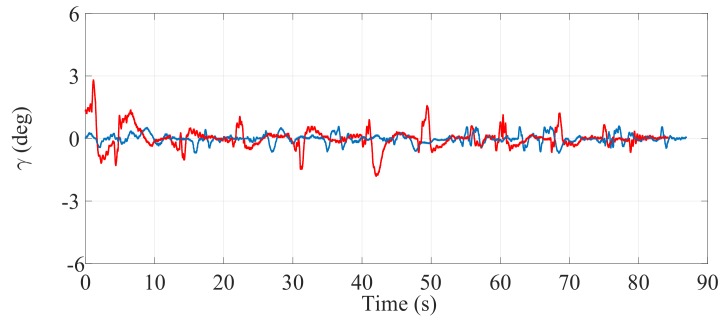
Inclination of the SCMS frame (γ) when the optimization is carried out with a α equal to zero (—) and when the optimization is not considered (—).

**Figure 22:**
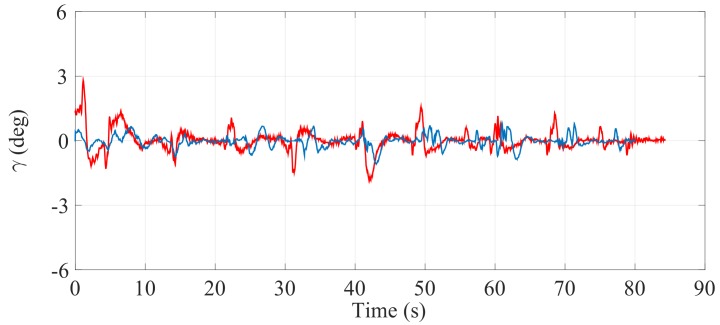
Inclination of the SCMS frame (γ) when the optimization is carried out with a α equal to 0.5 (—) and when the optimization is not considered (—).

**Figure 23:**
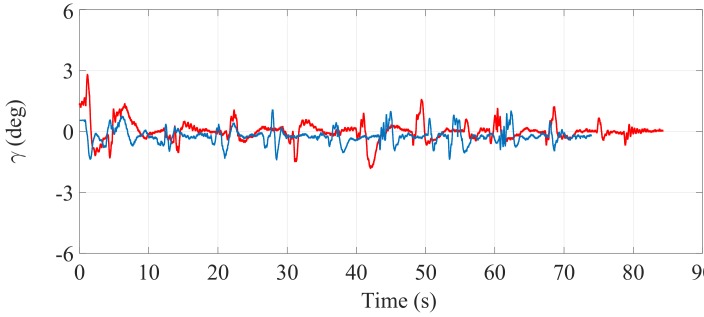
Inclination of the SCMS frame (γ) when the optimization is carried out with a α equal to one (—) and when the optimization is not considered (—).

Figure 21, Figure 22 and Figure 23 illustrate the experimental measurements of the inclination angle (γ) during the first stair-climbing process (Stair-Climbing Mode 1). These figures show a comparison between the inclination obtained when the desired trajectory profiles ($P^{*}$) that the SCMS will track are and are not optimized (in the first case, the methodology proposed in this paper is applied to optimize $P^{*}$, while in the second case, it is not applied and the profiles are not, therefore, optimized). When the optimization is carried out and the α of the cost function (32) is equal to zero, the variation in inclination is less than 0.7° (see Figure 21), signifying that an excellent control posture is achieved, which is associated with greater comfort and safety for the user. When the optimization is carried out with α equal to 0.5, the variation in inclination is less than 1.1° (see Figure 22), signifying that a good control posture is also achieved. When the optimization is carried out with α equal to 1, the variation in inclination is increased (less than 1.4°). However, note that the time spent on the climbing process is notably minimized (see Figure 23).

**Figure 24:**
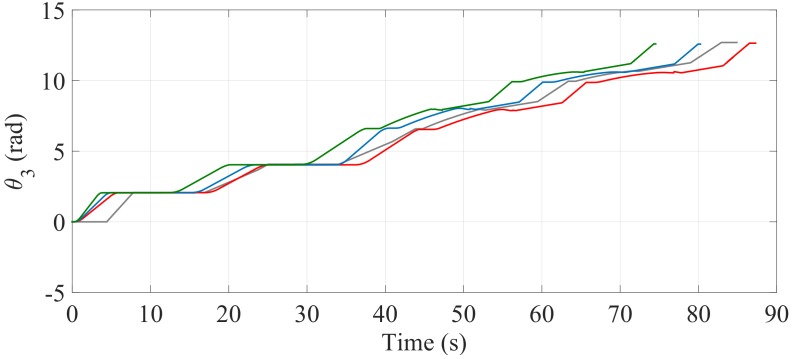
Behavior of the angular position of the driving wheels ($\theta_{3}$) when the optimization is carried out with α=0 (—), α=0.5 (—) and α=1 (—) and when the optimization is not considered (—).

**Figure 25:**
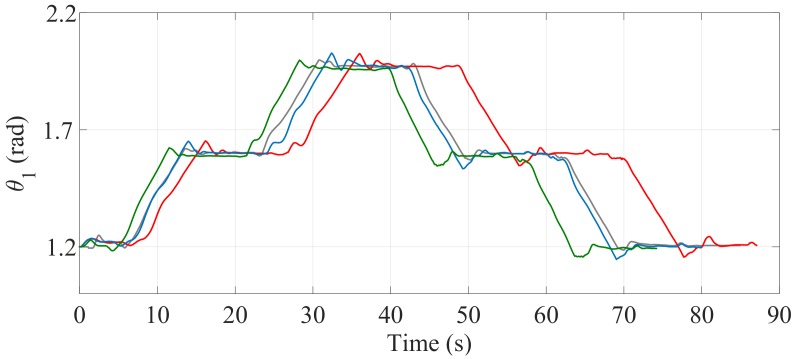
Behavior of $\theta_{1}$ when the optimization is carried out with α=0 (—), α=0.5 (—) and α=1 (—) and when the optimization is not considered (—).

Figure 24 and Figure 25 show the comparisons of the angular position of the driving wheels ($\theta_{3}$) and the angular position of the joint connecting the SCMS frame ($\theta_{1}$), respectively. The comparisons are also made when the desired trajectory profiles ($P^{*}$) are and are not optimized. In this climbing mode, the responsibility for the maintenance of the vertical position of the SCMS frame is entirely supported by the linear actuator related to $\theta_{1}$. This climbing strategy allows the angular position ($\theta_{2}$) to be kept constant during the stair climbing task ($\theta_{2}=1.1$ rad).

**Figure 26:**
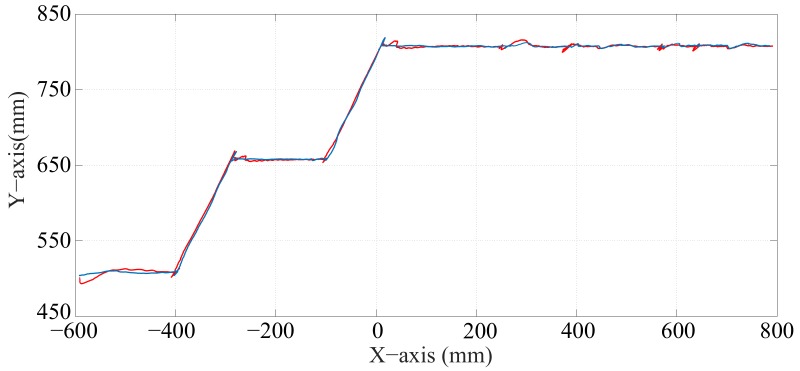
Path of the center of mass ($P_{g}$) when the optimization is carried out with α equal to zero (—) and when the optimization is not considered (—).

Figure 26 shows the position of $P_{g}$ when the optimization is carried out with α equal to zero and when it is not considered. Note that in the first case there is only a small deviation in the trajectory, which is barely perceived by the passenger. The results obtained from these stair-climbing experiments are shown in Table 6.

#### 5.2.2. Second Experiment

**Figure 27:**
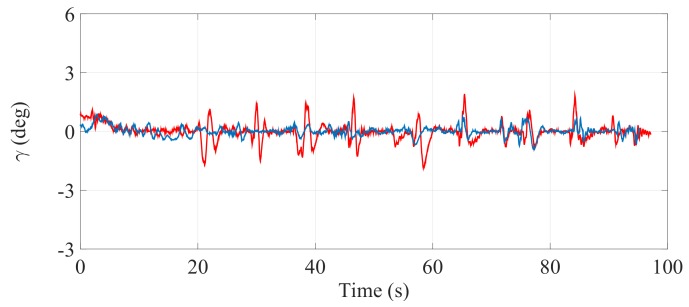
Inclination of the SCMS frame (γ) when the optimization is carried out with a α equal to zero (—) and when the optimization is not considered (—).

**Figure 28:**
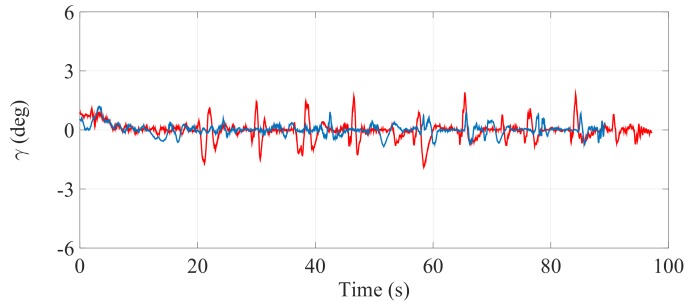
Inclination of the SCMS frame (γ) when the optimization is carried out with a α equal to 0.5 (—) and when the optimization is not considered (—).

**Figure 29:**
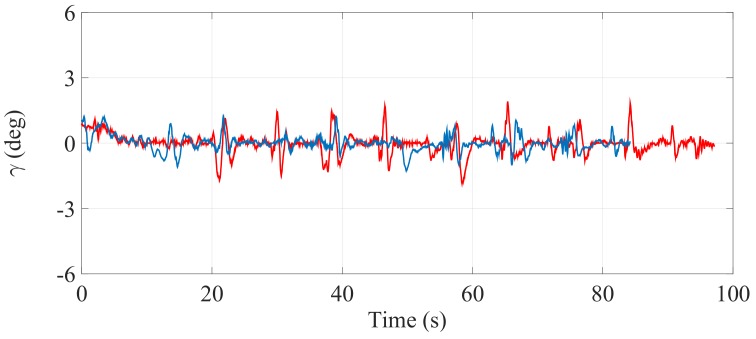
Inclination of the SCMS frame (γ) when the optimization is carried out with a α equal to one (—) and when the optimization is not considered (—).

Figure 27, Figure 28 and Figure 29 illustrate the experimental measurements of the inclination angle (γ) during the second stair-climbing process (Stair-Climbing Mode 2). These figures also show a comparison between the inclination obtained when the desired trajectory profiles ($P^{*}$) that the SCMS will track are and are not optimized. In this stair-climbing mode, the optimization is carried out with α equal to 0, 0.5 and 1. In the first case, the variation in inclination is relatively small (less than 0.82°), signifying that an excellent control posture is also achieved (see Figure 27). In the second case, the variation in inclination is less than 1.2° (see Figure 28). When α is equal to 1one, the variation in inclination is increased (less than 1.4°). However, note that the time spent on the climbing process is also notably minimized (see Figure 29).

**Figure 30:**
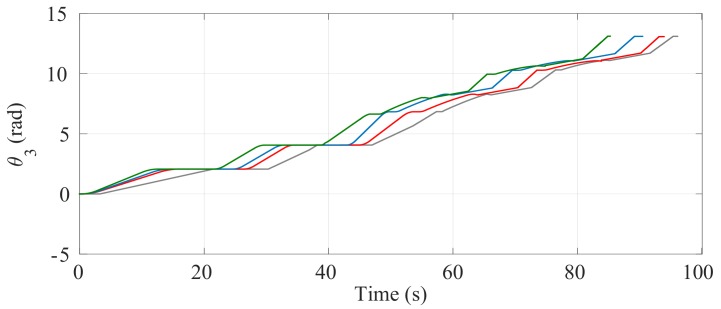
Behavior of the angular position of the driving wheels ($\theta_{3}$) when the path is optimized with α=0 (—), α=0.5 (—) and α=1 (—) and when the optimization is not considered (—).

**Figure 31:**
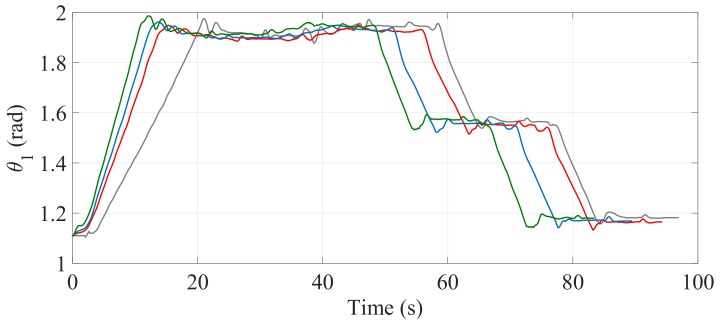
Behavior of the angular position ($\theta_{1}$) when the path is optimized with α=0 (—), α=0.5 (—) and α=1 (—) and when the optimization is not considered (—).

**Figure 32:**
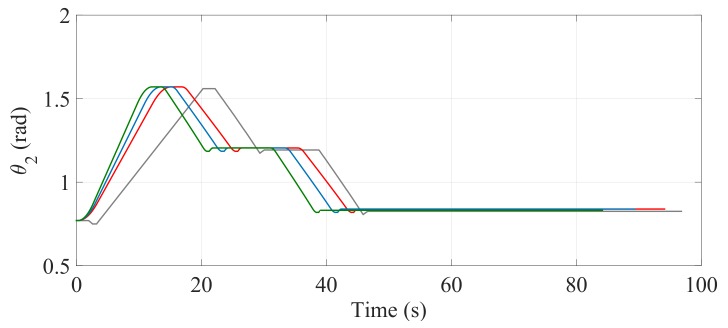
Behavior of the angular position ($\theta_{2}$) when the path is optimized with α=0 (—), α=0.5 (—) and α=1 (—) and when the optimization is not considered (—).

The comparisons of the angular position of $\theta_{3}$, $\theta_{1}$ and $\theta_{2}$ are shown in Figure 30, Figure 31 and Figure 32, respectively. Unlike the first climbing strategy, in this strategy the responsibility for the maintenance of the vertical position of the SCMS frame is supported by both the linear actuators related to these angles ($\theta_{1}$ and $\theta_{2}$).

**Figure 33:**
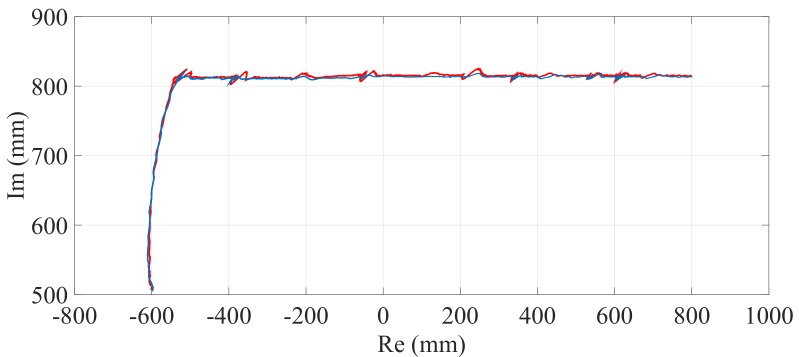
Path of the center of mass ($P_{g}$) when the optimization is carried out with α equal to zero (—) and when the optimization is not considered (—).

Figure 33 shows the position of $P_{g}$ when the optimization is carried out with α equal to zero and when it is not considered. As before, observe that when the optimization is considered there is a small deviation in the trajectory (note the regularity of the position of the center of mass during the maneuver). The results obtained after carrying out these stair-climbing experiments are shown in Table 7.

## 6. Conclusions

In this paper, we have developed an advanced trajectory generator with which to surpass those architectural barriers associated with climbing stairs. We have proposed a trajectory optimization technique that is used to improve the operation performance of the SCMS during the stair-climbing process. Two optimal reference trajectories (two different climbing strategies) for the platform carrying a user have been generated before confronting the architectural barriers. They have been generated by considering the information about stair geometry. This has allowed the control problem to be simplified, the time needed to climb/descend staircases to be reduced and the user’s safety and comfort to be improved.

The proposed technique makes it possible to minimize a cost function when subjected to the most important constraints inherent to the system behavior, such as the geometry of the architectural barrier, its reconfigurable nature, comfort and limits as regards actuator torques and speeds. Two sets of experiments were carried out using different time-comfort combinations and different climbing strategies in order to evaluate the proposed methodology when the autonomous SCMS surpasses staircases. The results show that this technique can be successfully used to generate optimal trajectories under various constraints inherent in the whole system. When the α of the cost function is equal to zero, the trajectory tracking is successfully achieved and a comfortable motion is generated. When the α is equal to one, the trajectory tracking is fairly good and the time spent on the climbing process decreases. The work presented in this paper once again demonstrates the feasibility and advantages of the proposed concept. When compared with other SCMSs, our prototype is characterized by the fact that it can climb up and down stairs of different sizes, including those with geometric disturbances, and maintain a stable equilibrium on the stair at all times without assistance.

## Figures and Tables

**Table 1:** Initial expressions that define the current position of the SCMS.

Configurations	Expressions	
1 (see Figure 11a)	$P_{g}=f\left(\theta_{3}\right)+l_{6}e^{j(\gamma +\frac{\pi }{2}+\mu_{6})}+l_{4}e^{j(\gamma +\frac{3\pi }{2}-\theta_{2})}+l_{5}e^{j(\gamma +\frac{\pi }{2}+\mu_{5})}$	(1)
	$P_{g}=f\left(\theta_{4}\right)+l_{1}e^{j(\gamma +\frac{\pi }{2}+\mu_{1})}-l_{3}e^{j(\gamma +\frac{\pi }{2}+\theta_{1})}+l_{5}e^{j(\gamma +\frac{\pi }{2}+\mu_{5})}$	(2)
2 (see Figure 11b)	$P_{g}=P_{C2}+z_{2}e^{j(\gamma +\frac{\pi }{2}-\delta_{2})}+l_{6}e^{j(\gamma +\frac{\pi }{2}+\mu_{6})}+l_{4}e^{j(\gamma +\frac{3\pi }{2}-\theta_{2})}+l_{5}e^{j(\gamma +\frac{\pi }{2}+\mu_{5})}$	(3)
	Equation (2)	
3 (see Figure 11c)	Equation (1)	
	$P_{g}=P_{C1}+z_{1}e^{j(\gamma +\frac{\pi }{2}-\delta_{1})}+l_{1}e^{j(\gamma +\frac{\pi }{2}+\mu_{1})}-l_{3}e^{j(\gamma +\frac{\pi }{2}+\theta_{1})}+l_{5}e^{j(\gamma +\frac{\pi }{2}+\mu_{5})}$	(4)
4 (see Figure 11d)	Equation (3)	
	Equation (4)	

**Table 2:** Terms of differential equations of the implicit Jacobian for the four configurations (where $\sin\left(\cdot \right)\equiv S_{\left(\cdot \right)}$ and $\cos\left(\cdot \right)\equiv C_{\left(\cdot \right)}$).

*k*	$\frac{\partial F_{k}}{\partial \gamma }$	$\frac{\partial F_{k}}{\partial \theta_{1}}$	$\frac{\partial F_{k}}{\partial \theta_{2}}$	*g*${}_{k}$($q,\dot{q}$)
1	$l_{1}S_{\left(\gamma +\mu_{1}\right)}-l_{6}S_{\left(\gamma +\mu_{6}\right)}+l_{4}S_{\left(\gamma -\theta_{2}\right)}-l_{3}S_{\left(\gamma +\theta_{1}\right)}$	$-l_{3}S_{\left(\gamma +\theta_{1}\right)}$	$-l_{4}S_{\left(\gamma -\theta_{2}\right)}$	
2	$l_{1}S_{\left(\gamma +\mu_{1}\right)}-l_{6}S_{\left(\gamma +\mu_{6}\right)}+l_{4}S_{\left(\gamma -\theta_{2}\right)}-l_{3}S_{\left(\gamma +\theta_{1}\right)}-z_{2}S_{\left(\gamma -\delta_{2}\right)}$	$-l_{3}S_{\left(\gamma +\theta_{1}\right)}$	$-l_{4}S_{\left(\gamma -\theta_{2}\right)}$	$+C_{\left(\gamma -\delta_{2}\right)}{\dot{z}}_{2}$
3	$l_{1}S_{\left(\gamma +\mu_{1}\right)}-l_{6}S_{\left(\gamma +\mu_{6}\right)}+l_{4}S_{\left(\gamma -\theta_{2}\right)}-l_{3}S_{\left(\gamma +\theta_{1}\right)}+z_{1}S_{\left(\gamma -\delta_{1}\right)}$	$-l_{3}S_{\left(\gamma +\theta_{1}\right)}$	$-l_{4}S_{\left(\gamma -\theta_{2}\right)}$	$-C_{\left(\gamma -\delta_{1}\right)}{\dot{z}}_{1}$
4	$l_{1}S_{\left(\gamma +\mu_{1}\right)}-l_{6}S_{\left(\gamma +\mu_{6}\right)}+l_{4}S_{\left(\gamma -\theta_{2}\right)}-l_{3}S_{\left(\gamma +\theta_{1}\right)}+z_{1}S_{\left(\gamma -\delta_{1}\right)}$	$-l_{3}S_{\left(\gamma +\theta_{1}\right)}$	$-l_{4}S_{\left(\gamma -\theta_{2}\right)}$	$-C_{\left(\gamma -\delta_{1}\right)}{\dot{z}}_{1}$
	$-z_{2}S_{\left(\gamma -\delta_{2}\right)}$			$+C_{\left(\gamma -\delta_{2}\right)}{\dot{z}}_{2}$

**Table 3:** Specifications of the SCMS.

Variable	Value
User’s weight	70 kg
Vehicle weight	60 kg
Height steps	150 mm
Wide steps	300 mm
Sampling time	15 mm

**Table 4:** Dimensions of the SCMS’s links.

Variable	Value
$l_{1}$	270 mm
$l_{3}$	410 mm
$l_{4}$	420 mm
$l_{5}$	323 mm
$l_{6}$	390 mm
$\delta_{i}$	35 deg
$r_{wheel}$	100 mm

**Table 5:** Specifications of the motors and actuators.

Variable and Value
$\gamma_{mim}=10{}^{\circ}$
$\gamma_{max}=-10{}^{\circ}$
${\dot{\gamma }}_{max}=\pm 0.22{}^{\circ}$/s
${\ddot{\gamma }}_{max}=\pm 0.1{}^{\circ}$/s${}^{2}$
$\theta_{1mim}=1.02$ rad
$\theta_{2mim}=0.7$ rad
$\theta_{1max}=2.32$ rad
$\theta_{2max}=1.97$ rad
${\dot{\theta }}_{1max}={\dot{\theta }}_{2max}=\pm 0.12$ rad/s
${\ddot{\theta }}_{1max}={\ddot{\theta }}_{2max}=\pm 0.03$ rad/s${}^{2}$
${\dot{\theta }}_{3max}=\pm 4$ rad/s
${\ddot{\theta }}_{3max}=\pm 0.24$ rad/s${}^{2}$
$z_{1max}=z_{2max}=260$ mm
${\dot{z}}_{1max}={\dot{z}}_{2max}=\pm 33$ mm/s
${\ddot{z}}_{1max}={\ddot{z}}_{2max}=\pm 0.6$ mm/s${}^{2}$

**Table 6:** Results obtained.

α	Climbing Time (s)	Comfort (Std)	Algorithm Execution Time (s)
0	86	0.0019	2.6
0.5	80	0.0032	3.1
1	74	0.0047	4.0
No optimization	85	0.0053	

**Table 7:** Results obtained.

α	Climbing Time (s)	Comfort (Std)	Algorithm Execution Time (s)
0	95.2	0.0021	1.1
0.5	90.0	0.0033	1.9
1	84.3	0.0042	3.6
No optimization	97.3	0.0062	

## Supplementary Materials

The following are available online at http://www.mdpi.com/1424-8220/17/11/2608/s1: Video S1: Climbing process of a three-step staircase; Video S2: Descending process of a three-step staircase.
